# University Students with Autism: The Social and Academic Experiences of University in the UK

**DOI:** 10.1007/s10803-018-3741-4

**Published:** 2018-09-01

**Authors:** Emine Gurbuz, Mary Hanley, Deborah M. Riby

**Affiliations:** 1 Department of Psychology, Durham University, South Road, Durham, DH1 3LE UK; 2 Centre for Developmental Disorders, Durham University, Durham, UK

**Keywords:** Autism, University, Education

## Abstract

The number of university students with autism is increasing, and it is crucial that these students can access adequate support. An online questionnaire was completed by 26 autistic students and 158 non-autistic students enrolled at UK universities to investigate social and academic experiences. Autistic students self-reported significant challenges and more mental health difficulties than non-autistic students. Significant challenges focused on the social components of university life, including social skills, social support opportunities, and levels of ASD awareness from others. Many strengths were also reported regarding academic skills of autistic university students. Importantly, there were more thoughts of withdrawal by the students with autism highlighting the need for support. These data can inform university student support services.

Autism Spectrum Disorders (ASDs) are complex neurodevelopmental disorders with lifelong impacts on social communication, alongside the presence of repetitive and restrictive behaviours (American Psychiatric Association 2013). ASD can occur with, or without, intellectual disability and cognitively able autistic^1^ individuals can still experience a number of social challenges that map on to the ASD diagnostic criteria (Eaves and Ho 2008; Bellini 2004). It is important to understand the life experiences of university students with autism to best appreciate how to provide support and develop opportunities to increase both societal engagement and quality of life (Van Heijst and Geurts 2015).

Research has shown that employment rates for autistic adults are as low as 4.1% (Taylor and Seltzer 2011). Furthermore, fewer autistic individuals are likely to continue into further or higher education (Shattuck et al. 2012). However, this picture is changing and this is partly due to investment in equality and diversity programmes and widening participation agendas. The number of students with autism completing a higher education qualification is increasing; in the US the number of autistic students at University is between 0.7 and 1.9% of the student population (White et al. 2011) and in the UK rates are reported slightly higher having increased from 1.8% in 2004 to 2.4% of the student population in 2008 (Macleod and Green 2009). These numbers are expected to have increased even further since these data became available and therefore there is a timely need to consider the specific requirements of autistic students (Friedman et al. 2013). This becomes especially important given evidence that less than 40% of autistic students successfully complete their studies (Vanbergeijk et al. 2008; Newman et al. 2011).

## Social Aspects of University for Autistic Students

There are several reasons why students with autism may find University life challenging, and more so than students without autism, especially considering combined social and academic demands. Three systematic reviews of research involving autistic University students all reported widespread social challenges (e.g. lack of social participation) and increased mental health concerns (e.g. stress, anxiety, and depression), with a further emphasize on the lack of support targeted towards non-academic issues (Gelbar et al. 2014; Anderson et al. 2017b; Jansen et al. 2018). There was an array of social challenges reported that included generalised difficulties with social skills, plus stress and anxiety in social situations (Accardo 2017), difficulties making friends (Gelbar et al. 2015; Jackson et al. 2018), problems managing emotions, self-determination (White et al. 2016), and self-advocacy difficulties (Elias and White 2018).

The reported social challenges could be related to the core deficits associated with ASD (e.g. including the role of theory of mind in understanding their peers) and therefore it is important to interpret the findings within a wider conceptual theoretical framework of autism (Gobbo and Shmulsky 2014). Indeed at the very core of ASD are the social and communicative difficulties that individuals experience throughout development; for example, difficulties with interpersonal skills, self-regulation, lower self-esteem, and a possible atypical social motivation that may impact upon learning social expertise from an early age (Myles and Simpson 2002; Dijkhuis et al. 2017; Matthews et al. 2015; White et al. 2016; Chevalier et al. 2012). Moreover, coping with independent living and new routines while adapting to a large number of new challenges, can also feed into difficulties for these students (Vincent et al. 2017; Jackson et al. 2018; Van Hees et al. 2015). As a result of this constellation of challenges, it has been reported that autistic students can experience heightened social isolation, loneliness, bullying, and stigmatization compared to their peers (Vanbergeijk et al. 2008; Madriaga et al. 2010; Gelbar et al. 2014). Moreover, depression and anxiety are reported as the most common mental health challenges for young adults with autism (Lugnegard et al. 2011; Volkmar et al. 2017) and these could be seen as both a consequence and a contributor to the issues noted above (Accardo 2017).

While some of these issues appear negative in nature, students with autism have several personal qualities that could help them in social situations when commencing University. Van Hees et al. (2015) interviewed 23 autistic university students who reported sincerity, fairness, and willingness to listen to others as personal strengths. Furthermore, some autistic students perceive the new and challenging social situation of University life as an opportunity to try and test their personal abilities, which indicates a very positive and flexible approach (Vincent et al. 2017). Therefore understanding how to capitalise on these strengths is crucial.

The previous studies identify the social aspects of university as particularly challenging for students with autism and it has therefore been proposed that providing appropriate social support is essential (Zeedy et al. 2016; Kuder and Accardo 2018). Furthermore, such support can have a positive consequence of improving quality of life and improving academic outcomes (Tobin et al. 2014) and it should incorporate the strengths mentioned above. Therefore, there is a timely need to increase the evidence-base in order to provide the most relevant support networks and capture insights from students in the UK.

## Academic Aspects of University for Autistic Students

According to a recent study, 48% of autistic university students were happy with their academic workload and considered themselves academically successful (Jackson et al. 2018; Gelbar et al. 2015). However self-reported experiences of autistic students have indicated that information processing speed, time management, group work, presentations, motivation to study, following lectures, and asking questions can all be significant challenges (Macleod and Green 2009; Van Hees et al. 2015; Anderson et al. 2017a; Jansen et al. 2018; White et al. 2016). These academic challenges may be linked to ASD related issues such as executive function abilities and weak central coherence (Gobbo and Shmulsky 2014; Vanbergeijk et al. 2008), as well as linking to some of the social aspects mentioned above (e.g. group work). In particular, switching from one task to another, prioritising knowledge for a specific assessment, and monitoring progress are all important skills. Touching on these issue, executive function training has recently been suggested as a potential target for intervention and support by the parents of university students with autism (Elias and White 2018).

Autistic students have reported that structure in academic settings, concrete instructions and smaller assignments helped them to deal with some of the academic challenges (Cai and Richdale 2016; Knott and Taylor 2014). They also mentioned many strengths such as proficient memory skills, a focus in detail, original and creative thoughts, passionate interests, the desire to acquire accurate knowledge, and adherence to rules when clear structure is provided. These skills can positively impact academic experiences and outcomes for autistic students (Anderson et al. 2017a; Gobbo and Shmulsky 2014; Van Hees et al. 2015; Drake 2014). Intervention and support mechanisms should capitalise on such competencies.

## Other ASD-Related Issues

Of course the challenges for students with autism are unlikely to be isolated to social and academic issues and additional challenges may be associated with sensory processing, fine-motor skills, and intolerance to changing routines, to mention a few (Vanbergeijk et al. 2008; Gelbar et al. 2014; Anderson et al. 2017a; Morrison et al. 2009). These ASD-related issues could influence the ability to navigate social environments, to adapt to new and fluctuating routines, to manage daily living activities, and therefore further determine well-being (Volkmar et al. 2017). In addition, both environmental and personal factors can influence students. For example, a lack of understanding and appreciation of differences among students, stigmatization and discriminatory practices on campus could prevent students with autism from disclosing their diagnosis and subsequently seeking support (Cox et al. 2017; Vincent et al. 2017; Sarrett 2018). Taking a comprehensive approach across domains of potential need, and considering strengths as well as challenges, is crucial to develop appropriate interventions and support.

## Current Study

The first-person accounts of students with autism have provided valuable insights into their University experiences (Gelbar et al. 2014; Vincent et al. 2017; Sarrett 2018). However, the majority of existing evidence comes from academics (Gobbo and Shmulsky 2014), or family members (Cai and Richdale 2016), rather than directly from autistic students. Published studies that have involved students have either had exceptionally small sample sizes (n = 5; White et al. 2016) or have been entirely qualitative in nature (Gelbar et al. 2014). To date there have been three quantitative questionnaire studies; two from the US (Gelbar et al. 2015; Jackson et al. 2018) and one from Australia (Anderson et al. 2017a), but none from the UK. Only two studies have combined qualitative and quantitative measures, both in the US (White et al. 2016; Accardo et al. 2018). None of the existing published studies have included students without autism as a comparison to know whether the issues and challenges are heightened, reduced, or similar in nature, for students with and without autism. Therefore, the current study is the first to (i) explore the first-hand social and academic experiences of university students with autism in the UK using a systematic approach including both qualitative and quantitative data, and (ii) include both students with autism and those without autism for comparison (age and study-matched typically developing students; non-autistic group). The current study aims to (i) understand self-reported social challenges as well as social strengths for students with and without autism, (ii) understand self-reported academic challenges as well as potential academic strengths for students with and without autism, (3) understand the formal (professional) and informal support received and reported by autistic students, and (4) understand self-reflections about having autism and being a university student (including opinions of awareness and acceptance of ASD by others). Based on the existing evidence, we hypothesize that students with autism will report more social challenges (and fewer social strengths) than their non-autistic peers, alongside more prevalent mental health issues. We also expect autistic students to self-report potential academic strengths, even alongside social needs. It is expected that students with autism will raise issues with the availability of specialised support, especially in social domains. Lastly, the students will mention identity and disclosure issues associated with having autism and being a university student.

## Method

### Participants

Twenty-six students with autism (14 male, 10 female, 2 other) and 158 typically developing (non-autistic) students (51 male, 99 female, 3 other; 5 missing data) participated in completing the online questionnaire. The autistic students self-reported their age of diagnosis and over 70% reported that this occurred after 16-years of age. There was no difference between groups in the mean age of the students at the time of participating in the study (*t* (182) = 1.701, *p* < 0.091). All participants were currently enrolled in higher education and studying at a university in the UK (across a variety of Universities, not named for anonymity and confidentiality of participants). For the sample as a whole, 50% were undergraduate students, 23% were studying for a Masters degree, and the remainder were studying at PhD level. For the students with autism, 89% were studying for a full time degree, whereas 11% were studying part time. For the non-autistic students, 91% were studying full time and 9% were studying on a part time basis. Across groups, most students were studying science subjects, followed by social science or health subjects, and then arts and humanities. Table 1 presents the demographic data obtained from autistic and non-autistic students.

**Table 1:** Demographics of the student groups with and without autism

Variables	Categories	ASD (%)	TD (%)
Sex	Male	53.8	33.3
	Female	38.5	64.7
	Others	7.7	1.9
	Did not report	0	3.2
Age (mean and SD in years)		26.35 (10.02)	23.96 (10.54)
Current level of study	Undergraduate	69.2	46.2
	Masters	19.2	24.1
	PhD	11.5	27.8
	Other	0	1.9
Type of study	Arts and humanities	11.5	19
	Science	65.4	43
	Social Science and Health	23.1	36.7
Mental health diagnosis	Anxiety/depression	46	14
	Other	8	3
	Total	54	17

Participants were asked whether they had a current mental health diagnosis (for example depression or anxiety). 54% of students with autism self-reported a diagnosed mental health condition (in addition to having an autism diagnosis), with anxiety and depression most commonly specified (46%). In comparison, 17% of non-autistic students self-reported a mental health diagnosis (14% for anxiety and depression; see Table 1).

### Materials

To understand the social and academic experiences of autistic students an online questionnaire was developed. After reviewing the literature, themes and issues emerged as particularly relevant to address, such as social skills, social motivation, isolation and loneliness, academic challenges, and adaptation to university life (Anderson et al. 2017b; Chevallier et al. 2012; Jackson et al. 2018). Therefore, we looked to relevant items from existing validated questionnaires on these issues, such as the Friendship Motivation Questionnaire showing a good test–retest reliability (*r* = 0.7) and internal consistency (Cronbach’s alpha = 0.75;Richard and Schneider 2005), UCLA Loneliness Scale with an internal consistency ranging from α = 0.89 to 0.94 (version 3; Russell 1996), and Rasch-type Loneliness Scale (De Jong-Gierveld 1985) with a reliability range of α = 0.81–0.95 (De Jong Gierveld and Van Tilburg 2010). Two other questionnaires developed for students; Student Adaptation to College Questionnaire (Baker and Siryk 1989) proving a high internal consistency (α > 0.80; Beyers and Goossens 2002) and the Survey of Current and Former College Students with autism (Gelbar et al. 2015) were reviewed for relevant items to be used in the current study. We adapted the wording of some questions to be culturally appropriate for a UK sample (e.g. changing ‘college’ to ‘university’). We also developed new questionnaire items on social motivation as this appeared as a key issue in previous studies but could not be fully captured with the existing measures.

The final 57 items that were used were Likert-scale questions answered on a 5-point scale from strongly disagree “5” to strongly agree “1”. They included questions about social functioning at University “I often feel I am involved in socializing with others”, social skills “I often find it difficult to socialize with others”, social motivation “I often get excited when I see an opportunity for meeting a new person I like”, motivation for friendship “I think that having friends does not bring much to my life”, academic functioning “I am enjoying my academic work”, satisfaction about academic performance “I am satisfied with the level at which I am performing academically”, and adaptations to the current institution “I have thoughts of withdrawing from my institution/course”.

In order to further capture some of these challenges, together with the strengths of students with autism, we included 7 open-ended questions where autistic students provided first-hand accounts of their experiences at university (see Table 2). These questions probed the support received at university, social and academic experiences as an autistic student, the biggest challenges encountered, helpful support they received, potential strengths, and finally the most important thing to know about being a university student with autism. These 7 questions would only be answered by the students with autism (and not by non-autistic students).

**Table 2:** Open-ended questions answered only by the autistic students

Questions	
1	What kind of additional support services and accommodation did you receive at your institution (e.g. college/university) due of your diagnosis of an ASD?
2	What do you think are the most important issues about the social experience of being a college/university student with an ASD?
3	What do you think are the most important issues about the academic experience of being a college/university student with an ASD?
4	What are the biggest challenges you face as a student with an ASD?
5	What has helped you most during the transition from secondary school to higher-education?
6	In your opinion, in what areas are you most successful as a student?
7	In your opinion, what is the most important thing we should know about being a student with an ASD in higher education?

The demographics section of the questionnaire included questions about age, gender, the name of the institution, the level of study, the subject of study, highest qualification achieved, enrolment type, nationality, relationship status, native language, age of diagnoses, other current diagnoses, anyone else in the family diagnosed with autism and their relationship to the participant, and finally the disclosure of autism to the current institution.

As a result, the final questionnaire consisted of both quantitative and qualitative items which was important for gaining rich and informative data. The entire questionnaire included 15 questions concerning sample demographics, 57 Likert-scale items and 7 open-ended questions probing social and academic experiences. The questionnaire was hosted via http://www.onlinesurveys.ac.uk.

### Recruitment and Procedure

Following ethics approval by the local ethics committee, individuals were approached via advertisements sent to a number of UK universities, University colleges, several organizations in UK working with autistic university students, and University Disability Services. The advertisement was also posted on a variety of social media outlets. The online link included information about the study, the consent form, and the online questionnaire. Autistic students self-reported a previous diagnosis of autism and non-autistic students reported no developmental issues. The questionnaire was anonymous and all answers were confidential. Participants were told they had the right to omit any questions they did not wish to answer and could withdraw their data from the study at any point until data analysis. At the end of the online questionnaire, participants were presented with the debrief page explaining the rationale and aim of the study and had the opportunity to enter a prize draw.

### Data Analysis Strategy

Principal Component Analysis (Jolliffe 2002) was applied to the 57 Likert-scale items to investigate the factor structure of the data. In order to extract components, the Kaiser (2016) criteria and scree tests were used, with Varimax rotations applied. Items that loaded on components with values > 0.40 were retained (Pedhazur and Schmelkin 1991). Finally, meaningful labels were given to each component.

The open-ended questions were analysed using data-driven thematic analysis to identify relevant themes for each question (Braun and Clark 2006). Initial codes were created by grouping the relevant data into smaller chunks and codes were then collated into potential themes. Twenty percent of the data was double-coded by an independent researcher and the inter-rater agreement level of 100% was obtained.

## Results

### PCA Analysis of Questionnaire Items

The check for multicollinearity as indicated by the Determinant was lower than 0.00001. The measure of sampling adequacy (Kaiser–Meyer–Olkin) was 0.83, which is recommended to be above 0.5. The Bartlett’s test of sphericity was significant, χ^2^ (1596) = 5264.061, *p* < 0.001, suggesting there were substantial relationships between variables and therefore it was possible to run the PCA analysis. Overall, the PCA was found suitable to run with 57 items.

Initial PCA with all the Likert-scale items resulted in 14 factors with Eigenvalues > 1, accounting for 69% of variance in the data. According to the scree plot, 4 factors were identified with 44% of variance explained. However, the last component had two items and only explained 4% of the total variance. Solutions for four, three, and two factors were each examined using Varimax rotations and items with loadings on components at or above 0.40 were extracted. A two-factor structure was chosen because each component explained the highest variance (24%, 10% respectively) and the third and fourth factors were not strong enough to be included as there were not enough items (Costello and Osborne 2005). In the final structure, 23 items loaded on the first component and 15 items loaded on the second component (see Table 3), 5 items loaded on both components. After investigating each cross-loading item conceptually, it was decided all 5 of them belong to the first component. Finally, the first component with 28 items in total was labelled as social functioning (α = 0.80), and the second component with 15 items was labelled as academic functioning (α = 0.89).

**Table 3:** PCA results and the percentage of students with and without autism who agreed with the corresponding questionnaire item

Factor	Items	Varimax rotations	ASD (%)	non-autistic (%)
1	Social functioning			
	I often find it difficult to socialize with others	0.787	70.5	34.1
	I often feel I am involved in socializing with others	0.785	12	57.4
	I often feel outgoing and friendly	0.751	20	50.3
	I often feel I don’t have any friends	0.687	65.4	19
	I often feel left out	0.674	69.3	33.6
	I often find it difficult to introduce myself to others	0.659	77	45.5
	I often feel I prefer to be alone	0.657	76	42
	I often feel shy	0.643	69.3	43.1
	I often get excited when I see an opportunity for meeting a new person I like	0.631	23	66.5
	I have the social skills to succeed at my institution	0.619	28	70
	I often feel that I have a lot in common with the people around me	0.611	23.1	47.5
	I think that having friends does not bring much to my life	0.598	19.2	3.8
	My motivation to have friends is the pleasure I get by talking to friends	0.568	69.2	88.6
	My motivation to have friends is the fun moments I have with friends	0.564	76.9	89.2
	I am involved in social activities at my institution	0.560	20	53.5
	I often feel willing to maintain my current friendships	0.558	64	83.5
	I often feel that my relationships with others are not meaningful	0.555	72	29.8
	I often feel alone	0.554	76.9	39.9
	I prefer to spend time in quiet places on campus	0.536	80	56.7
	My motivation to have friends is the fun of doing interesting things with friends	0.492	80	91.1
	I feel like people ignore me	0.479	60	21
	I often find it difficult to express my opinions to others	0.464	53.8	29.8
	My motivation to have friends is that friends make me feel better when I am sad	0.453	56	57.7
	I am better able to express myself with friends	0.406	42.3	47.7
	I have some good friends or acquaintances on my course with whom I can talk about any problems I may have	0.458	38.5	68.2
	I often feel like I get enough fun and enjoyment out of life	0.416	40	55.7
	I am having difficulty feeling at ease with other people on my course	0.440	68	26.2
	I feel I am very different from other students in ways that I don’t like	0.493	64	24.5
2	Academic functioning			
	I am enjoying my academic work	0.756	64	67.5
	I am quite satisfied with my academic situation at my institution	0.751	44	63.4
	I am adjusting well to my institution	0.737	40	82.7
	I feel confident that I will be able to deal in a satisfactory manner with future challenges here at my institution	0.691	38.4	76.6
	I have thoughts of withdrawing from my institution/course	0.667	56	15.3
	I get good grades	0.667	65.4	68.3
	I am pleased about my decision to go to higher education	0.650	76	89.8
	I really haven’t had much motivation for studying lately	0.643	60	30.6
	My academic goals and purposes are well defined	0.604	60	65
	I have the academic skills to succeed in my institution	0.584	70.9	78.4
	I am satisfied with the level at which I am performing academically	0.579	48	57.4
	I find it easy to focus when I am studying	0.518	32	37.6
	I have good study habits in terms of the time and activity I allocate to studying	0.497	50	50.4
	I find myself giving considerable thought to taking time off from my current institution and finishing later	0.482	40	12.7
	Lately I have been having doubts regarding the value of university education to me	0.477	28	14.7

The content of the social functioning component included socialisation, social skills, friendship, and social motivation. The content of the academic functioning component included academic skills (e.g. time management), satisfaction about the academic performance, and adaption to the institution (e.g. thoughts of withdrawing). Higher scores on each factor indicates better functioning. Compared to the non-autistic students, those with autism had significantly lower scores both in social (*p* < 0.001) and academic components (*p* < 0.001). The individual items that loaded on the social functioning component indicated that compared to non-autistic students, students with autism had more difficulties in socialising and making friends, they were less involved in social activities, they preferred to be alone, and they did not believe that their relationships with others were as meaningful. Nevertheless, they reported similar motivation to form friendships as their non-autistic peers (Table 3).

For the academic functioning component, students with autism reported enjoyment in their academic work, good academic grades, and they believed they had the necessary academic skills to succeed as much as other students without autism. On the other hand, they reported more adjustment problems, more frequent thoughts of withdrawal, and difficulties with academic motivation. Moreover, 35% of the autistic students reported to not feel confident to cope with the future challenges, compared to only 7% of non-autistic students (Table 3).

The correlation between social functioning score and academic functioning score was studied in each group (with some caution for sample size of the autism group where n = 26). The relationship between social and academic functioning was significant in both groups (non-autistic students *r* = 0.301, *p* = < 0.001; autistic students *r* = 0.589, *p* = 0.002).

### Thematic Analysis

Four main themes emerged from 7 open-ended questions answered by the autistic students; social functioning (1), academic functioning (2), ASD-related issues (3), and support and awareness of ASD by others (4). All of these themes contributed to the experience of autistic students (only the students with autism completed these questions). The following section discusses each sub-theme and provides student quotes to illustrate the theme / sub-theme. The numbers of students mentioned the corresponding sub-theme is provided in Table 4 alongside examples.

**Table 4:** Thematic analysis results including the frequency of themes and subthemes for the autistic students (*n* = 26)

Themes	Subthemes		*n*	Example quotes
Social functioning	Social skills	Socialisation/making friends Difficulty expressing yourself Self-advocacy and awareness of problems	23	“Knowing the right level of chilled/excited with friends… It is incredibly isolating and lonely, and for me personally, has been a very destructive process”
	Social activities	Organizing a life-work balance Unpredictable and anxiety provoking Hard to initiate and be involved Forced and not diverse Hard to find people with similar interests	17	“If you don’t drink or find clubs/bars/etc nightmarish, there are virtually no opportunities for you to socialise, especially if you’re also frightened off by societies like I am. I haven’t gone out and been social once in my entire first year here, and have talked to maybe a maximum of three other people on my course”
Academic functioning	Academic challenges	Need for guidance and clear instructions Not knowing how to pace Absorption in one subject Processing time Organizational skills Attention problems Group work and supervisor relationships Visualising abstract concepts/ Motivation/procrastination Critical/creative thinking Research/data analysis	12	“The temptation to delve into all the new and interesting academic information to hide from having to socialise. The urge to study ALL THE THINGS, ending up not covering enough material to a superficial level”
	Academic strengths	Academic and critical writing Ability to work long hours Understand complex ideas memory	20	“Academically, I came top on my course in terms of grades, so it’s hard to beat that. I don’t think I was the cleverest, nor the most innovative, but I have an excellent memory and was willing to just write whatever argument I thought the lecturer marking would want to see, and most of the time that was all that was required”
ASD related issues	Sensory overload and sensitivity to change	Noisy environments in the campus Harder to calm in new environments Hard to build routines	13	“The social activities can be unsettling as this is a change of routine. When I get settled into them, they are quite enjoyable, but it is working out how to fit in”
	Mental health challenges	High level of comorbidity Isolation/loneliness Daily activities being very stressful and anxiety eliciting	16	“The sheer quantity of new things (especially new people) causes brain overload and anxiety. I find many everyday activities more difficult and stressful than other people do, but this is not always obvious to those around me”
Support and Awareness of ASD	Suppor	Academic support Non-academic support Accommodations Personal strengths	16	“A strong confidence and ability to get on with things when I need to helped me the most during university”
	Awareness of ASD from others	Understanding their challenges/needs Heterogeneity among the students Stigmatization/different attitude	18	“I think society executives should have some form of awareness training about how to include people with ASD and be more accessible. Especially as joining societies may be the only way to meet people. Understand that people with ASD won’t necessarily ask for help when they need it, either because they don’t know how to or they don’t know that they should”

#### Theme: Social functioning

The questions regarding social functioning asked about the biggest challenges autistic students faced and the most important issues about the social experience of being an autistic student. As a result, two sub-themes were identified: social skills and social activities.

##### Social Skills

The students self-reported that they found it hard to initiate a social interaction, to express themselves to others, and to make new friends. They also mentioned that they felt anxious in social situations. As a result, 11 of the students (42% of the autism group) self-reported that they experienced social isolation and loneliness during their university years, though there was 58% of the students with autism who did not mention this as an issue (individual variability is evident throughout the data). Students commented on their biggest challenges as an autistic student:

Example “Socialising with people with different interests/personalities - but met people with similar interests so it wasn’t so bad.“

##### Social Activities

8 autistic students (31% of the autism group) reported that they found social activities forced, unnecessary, and not very diverse for people with different interests.

Example “I don’t like how there are seemingly hundreds of parties or other alcohol-consumption related events being thrown in your face every 10 min. I would be happy to not partake in the “social experience” and mind my own business but I can’t even walk to lectures without people trying to give me flyers or talk to me about some “very fun” thing they are trying to get people to do and appear to judge me for not being interested in their parties when I reject them.“

The other difficulties with engaging in social activities were their unpredictable and overwhelming nature. This is very important for students with autism due to difficulties with new environments and a new, or changing, routines which might further increase stress levels when engaging in social situations.

Example “Difficulty being included in social activities due to other people not understanding you need help being included in activities. Lots of stuff revolves around “going out” and drinking which is often too loud/crowded and may not want to drink. Also can be difficult to attend society events because not enough information is provided so you can’t prepare and it’s too scary.“

#### Theme: Academic functioning

Academic skills were investigated by asking about the most important issues and most successful academic experiences as an autistic student. These two questions resulted in two sub-themes; academic challenges and academic strengths.

##### Academic Challenges

Many students with autism reported similar challenges that influence their academic functioning. The most frequent challenges that were reported were the absorption in one subject at the cost of others (*n* = 8; 31%) which might lead to burnout in some circumstances as a small number of students self-reported that they did not know how to pace them self (*n* = 2; 8%) and a larger number self-reported that they often lack clear instructions of what was expected of them (*n* = 7; 27%).

Example “Knowing what’s expected of you. Lecturers take it for granted that you’ll understand what they’ve said or what they want, or that you’ll do something without being told to.“

The other issue that a small number of autistic students self-reported was perfectionism (*n* = 3; 12%) and as a result these students reported that they had a constant binary feeling of failure or success. They self-reported that it was hard to know whether the work they completed was good enough, which could also cause exhaustion.

Example “It is easy not to pace yourself. Partly I think I enjoyed my subject so was happy to spend more time than most studying, but also a pursuit for perfection or feeling that you failed is an unhealthy motivation. I think high grades are often taken to show you don’t need academic support, when really extremes on both sides (very high or very low marks) can flag extreme and unhealthy practices. So I think more support is necessary to ensure autistic students pace themselves and don’t burn out or feel that they have failed even if they have done well by more normal standards.“

Other academic challenges were working in groups, time management (e.g. procrastination), processing speed, organizational skills (e.g. including life-work balance), attentional skills, and motivation for studying or attending lectures.

Example “Course elements which require interacting with people when individual tasks could achieve the same purpose. For example, my programme contains a “Research Ethics” module in which all activities are group-based discussions and such, but this could just as easily be assessed with an individual piece of writing, and having this option for students with social or communication difficulties should be possible. I feel largely the administration ignores that these issues exist and/or feel that one should just have to put up with some discomfort from time to time.“

##### Academic Strengths

Alongside the academic challenges, students with autism self-reported that they felt they had a number of academic strengths. Students stated that they could study for long hours (n = 3; 12%), focus in detail on one subject (n = 4; 15%), and could use critical thinking and understand complex ideas (n = 4; 15%) and academic writing and research skills (n = 9; 35%). Naturally there was individual variability of the strengths that were mentioned.

Example “If I can get myself focused on a topic then I will go into much more detail and learn much more about it than other students.”

Interestingly, a small number of students mentioned that their difficulties could be underestimated due to their high academic grades, and indeed the social challenges may be masked by strong academic performance.

Example 1 “The academic side of things, for me, is easy. The biggest challenges are the social aspects I put above.”

Example 2 “…being expected to participate in activities/assessment formats which cause extreme distress e.g. oral presentations and lecturers being certain I will manage because my grades are good and downplay the anxiety I have about these types of assessments.”

#### Theme: ASD-related issues.

##### Sensory Overload and Sensitivity to Change

Individuals with autism can be sensitive to sensory stimuli in the environment and this may also impact their experiences at University. More than half of the autistic students reported issues with the sensory overload (hyper-sensitivity to sensory input) and noted that this affected both their academic performance and their motivation to participate in social events.

Example “Lectures and tutorials are noisy and crowded; I often become anxious and struggle to process the content above the background noise. The biggest challenge was being able to find a quiet place to work and revise (very distracted by noise) but I live near home so moved home during exam time.”

The other core feature of ASD that was reported by the students was sensitivity to changes in the routine. This could also be related to their need for clear structure on assessment and their reluctance to engage in new environments.

##### Mental Health Challenges

As previously noted, 54% of the current sample reported having mental health issues and many students (*n* = 10; 38%) reported difficulties navigating their social and academic world due to their mental health challenges. Please see Table 4 for example quotes.

#### Theme: Support and Awareness of ASD

The participants answered two broad questions to probe the support they received and what helped them the most in their transition to University life. Finally, participants were asked about the most important thing we should know about being a University student with autism. The responses to these questions resulted in two sub-themes of support and awareness of ASD by others.

##### Support

Autistic students (*n* = 16; 62%) reported that they received support from their institution and the types of support that were explicitly mentioned included mentoring (*n* = 6; 23%) disability services support (*n* = 4; 15%) and study tutors (*n* = 2; 8%). Moreover, accommodations such as exam allowances (e.g. extra time, extensions), use of a dictaphone or a note-taker in lectures, alternative assessments methods, and specialised rooms were provided for students. However, 5 students (19%) stated that they did not receive any support (even when requested) and 2 students (8%) reported that they did not ask for help or support thinking that it would not be useful (as illustrated in the example below):

Example “Nothing, but I have not requested or looked into receiving any additional support as I feel it’s largely unneeded. Most of the things that such services offer are probably of little help for my particular case. I don’t want help making friends for a rich social life, or special activities for the disabled, etc. I want to be able to manage my anxiety enough to function professionally (for which I have had out-of-university Cognitive Behavioural Therapy) and be left alone as much as possible beyond that.”

In addition to professional support, some students reported professional support at their institution, social support (e.g. family friends, partners) along with several personal qualities such as independence and confidence, which helped them in their transition from school to university (see Table 4 for more examples).

Example “I disliked secondary school because I never accepted that parents or teachers had authority over me, and I was never willing to obey unreasonable rules. I enjoyed the freedom to pursue a subject I was very interested in, and to be captain of my own ship.”

##### Awareness of ASD by others

12 students (46%) stated that society should better understand the challenges experienced by autistic individuals, especially in social situations, and approach them without being stigmatizing or patronising. Students also emphasized the importance of the diversity and heterogeneity among individuals with autism and they mentioned that they do not want to be perceived or treated differently (*n* = 5; 19%). Indeed awareness and training in ASD awareness were important to the views expressed by the students with autism and to encourage the involvement of autistic students and be more accessible.

Example “Every ASD person is different in how much attention and social enrichment they want or need. Everyone’s experience is different, and no two students will face the same difficulties. Understanding without being patronising is key, and the condition can be both a blessing and a curse at this level.”

## Discussion

The current study investigated the social and academic experiences of current university students with and without autism in the UK. The systematic analysis of qualitative and quantitative data indicated both social and academic challenges for students with autism compared to non-autistic students, supporting the core hypotheses and previously published research from students in the US (Gelbar et al. 2015; Anderson et al. 2017b; Jackson et al. 2018; Sarrett 2018). Moreover, high rates of self-reported mental health issues were evident and these were higher than those reported by non-autistic students. Several themes emerged from the qualitative data provided by the autistic students which supported the data from the Likert-scale items of the questionnaire. These additional qualitative insights provide rich illustrations of the experiences of students with autism at university. Indeed the current study is the first to combine insights from autistic and non-autistic students for direct comparison, while using both qualitative and quantitative data to understand University experiences in the UK.

The first aim of the study was to understand the social challenges and strengths of students with and without autism. Overall, the students with autism self-reported poorer social skills compared to non-autistic students. It was clear from the questionnaire items that the biggest challenges were difficulties with social interactions, loneliness, and lack of interpersonal skills, though there was naturally individual variability in the challenges that were reported. In fact, students with and without autism reported similar motivation levels for friendships such as pleasure talking to friends (70% in ASD; 88.5% in TD) and having fun times being with friends (77% in ASD; 89% in TD). The desire to form and maintain friendships in ASD supports previous studies (Sumiya et al. 2018; O’Hagan and Hebron 2017; Mazurek 2014). The research, however, showed that the autistic individuals might not put their knowledge about friendships into practice (Calder et al. 2013) due to broader social skill difficulties (Sedgewick et al. 2016). This could explain why 72% of the autism group did not find their relationships with others meaningful, and 66% reported having no friends. This is in line with the previous research suggesting social challenges (Gelbar et al. 2015; Knott and Taylor 2014; Cai and Richdale 2016; Tobin et al. 2014; Muller 2008). Supporting the quantitative data, the qualitative insights emphasised similar social challenges which could lead to social isolation and loneliness in some cases (Whitehouse et al. 2009) Indeed social activities were considered as overwhelming, unpredictable, and superficial. Importantly, there were large individual differences shown by the students with autism, with significant variation expressed in the desire for social interactions, and the degree of social challenges experienced. It is highly relevant that social participation is a central facet of the university life (Orsmond et al. 2013) and the current research suggests that providing further support for the social challenges experienced by some (but not necessarily all) autistic students could go some way to easing the transition from school to university (e.g. see Wehman et al. 2014). Considering the diverse social opportunities available to students would also be beneficial because many autistic adults show a motivation towards having friends, enjoy social activities (Sarrett 2018; Van Hees et al. 2015), and are willing to participate in social opportunities (Orsmond et al. 2004), therefore providing activities with more structure and a more diverse range of activities may facilitate higher engagement levels (and reduce the potential for social isolation; Sosnowy et al. 2018).

The second aim was to identify the self-reported academic strengths and challenges of university students with and without autism. Overall, the students with autism reported enjoyment in their academic work, said they received good academic grades, or had good study habits (e.g. academic focus) in line with previous research (Gelbar et al. 2015; Jackson et al. 2018). However, this positive experiences of academic life occurred together with some challenges and adaptation difficulties. A significant proportion of the autistic students reported that they had difficulty adjusting to their institution and 56% reported that they had considered withdrawing or taking a break from their studies, which was significantly more than reported by the non-autistic students. These issues for the students with autism could be explained by a lack of confidence in dealing with the future challenges, and difficulties finding the motivation to study (both issues reported in the current study by autistic students). The qualitative analysis included reports of ‘poorer’ academic functioning, difficulties working in groups, over-absorption in one subject, pursuit of perfection, reduced processing speed, time management difficulties and a lack of organizational skills. Some of these challenges have also reported been in previous studies (Van Hees et al. 2015; Knott and Taylor 2014) and this suggests that these may be consistent features for students with autism that warrant support for academic services within Universities (Barnhill 2016). Importantly, we need to consider the opportunity to capitalise on academic strengths of autistic students and these were self-reported in their research skills, written abilities, analytical thinking, understanding complex ideas, and an ambition to learn their subject of interest. Similar strengths and factors to promote success among autistic university students (e.g. self-determination) have been identified in previous studies (Drake 2014; Gobbo and Shmulsky 2014; Accardo 2017) and again it has been previously suggested that these strengths could be capitalised to enhance academic outcomes (Iovannone et al. 2003) but this approach has not yet been applied to higher-education students with autism. Taking into account personal strengths when developing support strategies would help students achieve their full academic potential.

Both social and academic challenges reported by the autistic students could derive from broader ASD-related issues. For instance, students with autism reported difficulties in building relationships with the supervisors and working in groups. Both these situations require social interaction skills. These two issues could be underlined by the core features of social communication and aspects of Theory of Mind difficulty associated with ASD (Marans et al. 2005). In a similar way, other ASD-related symptoms such as responses to overwhelming sensory stimuli, and sensitivity to changes in routine, could also influence the ability to adapt and navigate in social and academic environments at University (Grapel et al. 2015; Bodfish 1999). This is supported in the current study, where students with autism reported problems with adapting to changes in class and being overwhelmed by noisy and crowded lectures. So, while there will be significant individual differences, there is also a need to incorporate understanding of the core features of the autism spectrum into support for these students.

The third aim was to identify the support that students with autism received and explore how this could be improved. Even though students reported that they received professional support (e.g. study advisor, mentor, disability service), there were 6 students (23%) who did not receive any support. Equally, in some cases the offered support was entirely for academic functioning, but the main areas of need were non-academic in nature (e.g. social communication). With this in mind, some students reported the need for more expertise in understanding ASD and feeding this knowledge into planned support, which is also addressed in previous research (Ashbaugh et al. 2017). Many students commented on how it could currently be very difficult to ask for a help, or they would sometimes not recognize when they needed help. Therefore, it is important for people working with autistic students to be aware of potential difficulties with self-identifying issues and requesting support in a proactive manner. This requires an easily accessible support system with regularly scheduled support opportunities/meetings. A support group model with regular meetings integrated into the curriculum has previously been found to be efficient in improving both social and academic functioning of students with autism in the US, and reducing anxiety and depression (Hillier et al. 2017). This latter issue is crucial given the high self-reports of anxiety and depression in the current sample. So therefore, it is crucial for professional support staff within Universities to understand the cognitive and social experiences associated with a diagnosis of ASD and to use this understanding to help plan effective support (Rodgers and ofield 2018).

The autistic students self-reported a wide array of challenges and needs, indicating significant heterogeneity in their experiences and proficiencies. This heterogeneity adds a further challenge for support services. A personalized support system would be more beneficial to track individual needs and intervene accordingly. A recent pilot study with Australian university students with autism investigated the effect of specialized peer-mentoring with flexible and individualised support (Siew et al. 2017). The researchers found that students performed better academically and socially and they had higher retention rates. There was evidence of increased socialisation through new friendships, and a reduction in reports of communication difficulties (e.g. with both peers and University staff). An individualised support network allows the opportunity to also capitalise on strengths at an individual level. For example, some autistic students in the current study reported being independent, a good listener, and confident to get on tasks when needed and these competencies can contribute to better daily functioning (Van Hees et al. 2015) and could be further incorporated into practice and student support programmes (Lanou et al. 2012). For example, initial screening of individual strengths and competencies in autistic students could give insight into the best possible support strategies for more personalised and tailored intervention. However, the downside of this approach is the need to invest in expertise to complete evaluations and expert support staff with sufficient knowledge of ASD and this requires additional University resources (e.g staff, financial investment, time for student support).

Finally, autistic students reported the lack of awareness and a lack of acceptance of ASD. Many students highlighted that they felt their lecturers and other students did not have sufficient insight into their difficulties, or an acceptance of their differences. This may be a cause underlying, or leading to, social isolation or even bullying of students with autism (Pinder-Amaker 2014). A recent study showed that neurotypical observers were found to build negative and less favourable opinions of both children and adults with autism engaged in a social interaction (Sasson et al. 2017). Additionally, the observers reported reduced desire to develop future social relationships with autistic individuals. A lack of social acceptance may also feed into mental health difficulties experienced by those with autism, as reported here (see also Cage et al. 2018; Griffith et al. 2011). Sasson and colleagues (2017) reported that autistic adults who reported high rates of depression and who considered there to be a lack of autism acceptance, were more likely to display ‘camouflaging’ behaviour, which has been reported in another recent study as well (Lai et al. 2017). The constant struggle to look “normal” can lead to increased levels of anxiety and social withdrawal and therefore it is important to consider all of these issues in tandem with the aim of increasing awareness and appreciation of neurodiversity in higher education students, in order to create a more inclusive and supportive environment.

### Limitations and Future Directions

There are some limitations of the current study. The sample size of the non-autistic group was larger than the autism group, and indeed the sample size for the autistic students was relatively small. Nevertheless, the sample size of the autistic group was bigger than the average sample size of the previously published studies conducted with higher education students with autism outside the UK (*n* = 16; Anderson et al. 2017b). Future research should aim to include more students with autism across a wide range of UK institutions which would allow further exploration of individual differences in addition to the core issues studied here. The other limitation is the uneven representation of sex in each group as the TD group has a bigger female:male (2:1) ratio than the autism group (1:3). This is important because the reported needs and challenges could be related to sex differences rather than the ASD diagnosis. Therefore, future research should further explore the impact of sex on the experience of studying at University. This was not an aim of the current study but a future project focusing on sex differences in the social and academic experiences would be particularly useful for further investigating females with autism.

A further issue to reflect upon for the current study was that the ASD diagnoses, and the reporting of mental health difficulties, were self-reported. Confirmation of diagnosis would be beneficial in future research though this is a challenge for working across a number of educational institutions and accessing students in sufficient numbers. On the other hand, self-report measures have been increasingly used in research with autistic participants (e.g. anxiety and depression research, Williams 2010; personality research; Hesselmark et al. 2015) and these data is crucial for gaining insights into personal experiences and giving autistic adults a voice within society.

Given that the current study only looked at the self-reported experiences of students with and without autism, it is difficult to infer the underlying mechanisms behind the reported experiences (both challenges and proficiencies). There could be potential mechanisms, such as a role for executive function, emotion regulation, social motivation, and theory of mind (Gobbo and Shmulsky 2014; White et al. 2016) that underpin a number of the issues raised by autistic students in this study. Future research should measure these constructs using more direct and objective methods and correlate these with the reported University experiences for students with autism. In a similar vein, the literature would also benefit from longitudinal studies with follow-up measures to examine how social and academic experiences in higher education can influence outcomes for students with autism (e.g. employment, quality of life). In more clinical terms, the current study provides insights into the support needs of autistic students and newly developed support systems should focus on increasing awareness of ASD among staff and other students, while considering individual differences between students with autism, and trying to capitalise on potential strengths. With this in mind the aim is to provide the best possible support for both academic and social participation to enhance the likelihood of autistic students reaching their full potential.

## Conclusions

The current study was the first to compare the social and academic challenges and needs of age and study-matched students with and without autism in higher education in the UK providing both qualitative and quantitative data. The combination of demographic, quantitative and qualitative data provided further insight into the nature of self-reported social and academic experiences. The responses to open-ended questions indicated issues such as self-advocacy problems, vast heterogeneity in terms of proficiencies and challenges, and reported that autistic students felt there was a lack of awareness and acceptance of ASD. In order to promote a good transition to University, and in order help students with autism reach their full potential, all these factors should be considered in developing appropriate and effective interventions and support for autistic students.

## Footnotes

1 The term ‘autistic person’ is preferred by many individuals on the spectrum (Kenny et al. 2016). Therefore, we alternate between the use of this term and person-first language (‘person with autism’) throughout the paper in order to respect to wishes of all members of the community. Similar approaches have been adopted in existing published studies (Sedgewick et al. 2016).
